# The Natural Alkaloid Tryptanthrin Induces Apoptosis-like Death in *Leishmania* spp.

**DOI:** 10.3390/tropicalmed7060112

**Published:** 2022-06-20

**Authors:** Andreza R. Garcia, Yasmin P. G. Silva-Luiz, Celuta S. Alviano, Daniela S. Alviano, Alane B. Vermelho, Igor A. Rodrigues

**Affiliations:** 1 Graduate Program in Pharmaceutical Sciences, School of Pharmacy, Federal University of Rio de Janeiro, Rio de Janeiro 21941-902, Brazil; deh.raposo@yahoo.com.br; 2 Graduate Program in Science (Microbiology), Institute of Microbiology, Federal University of Rio de Janeiro, Rio de Janeiro 21941-902, Brazil; yasminpaulaluiz@gmail.com; 3 Department of General Microbiology, Institute of Microbiology, Federal University of Rio de Janeiro, Rio de Janeiro 21941-902, Brazil; alviano@micro.ufrj.br (C.S.A.); danialviano@micro.ufrj.br (D.S.A.); abvermelho@micro.ufrj.br (A.B.V.); 4 Department of Natural Products and Food, School of Pharmacy, CCS, Federal University of Rio de Janeiro, Rio de Janeiro 21941-902, Brazil

**Keywords:** tryptanthrin, alkaloid, apoptosis-like, antileishmanial activity, computational analysis

## Abstract

Leishmaniasis is a vector-borne disease against which there are no approved vaccines, and the treatment is based on highly toxic drugs. The alkaloids consist of a chemical class of natural nitrogen-containing substances with a long history of antileishmanial activity. The present study aimed at determining the antileishmanial activity and in silico pharmacokinetic and toxicological potentials of tryptanthrin alkaloid. The anti-*Leishmania amazonensis* and anti-*L. infantum* assays were performed against both promastigotes and intracellular amastigotes. Cellular viability was determined by parasites’ ability to grow (promastigotes) or differentiate (amastigotes) after incubation with tryptanthrin. The mechanisms of action were explored by mitochondrion dysfunction and apoptosis-like death evaluation. For the computational pharmacokinetics and toxicological analysis (ADMET), tryptanthrin was submitted to the PreADMET webserver. The alkaloid displayed anti-promastigote activity against *L. amazonensis* and *L. infantum* (IC_50_ = 11 and 8.0 μM, respectively). Tryptanthrin was active against intracellular amastigotes with IC_50_ values of 75 and 115 μM, respectively. Mitochondrial membrane depolarization was observed in tryptanthrin-treated promastigotes. In addition, parasites undergoing apoptosis-like death were detected after 18 h of exposure. In silico ADMET predictions revealed that tryptanthrin has pharmacokinetic and toxicological properties similar to miltefosine. The results presented herein demonstrate that tryptanthrin is an interesting drug candidate against leishmaniasis.

## 1. Introduction

*Leishmania* spp. are obligate intracellular protozoan parasites that invade and proliferate inside phagocytes, mainly macrophages. In the first step of infection, metacyclic promastigotes are transmitted during the bloodmeal of female phlebotomine sand flies and rapidly infect the host cells, where they differentiate into amastigotes. The last ones are the evolutive form responsible for the clinical manifestations of the so-called leishmaniasis in the vertebrate host [1]. The disease can evolve clinically as cutaneous or mucocutaneous lesions (tegumentary leishmaniasis) or as visceral injuries (visceral leishmaniasis) as a consequence of infection by dermotropic or viscerotropic species, respectively. Brazil is one of the few countries in which both clinical manifestations are endemic. In fact, in 2020, 16,056 and 1954 cases of cutaneous and visceral leishmaniasis were reported in this country, respectively [2].

Despite the efforts of several research groups to develop effective vaccines, none are available to fight human leishmaniasis [3]. In this scenario, leishmaniasis treatment still relies on the use of chemotherapeutic agents, mainly pentavalent antimony, amphotericin B (conventional or liposomal formulations), and miltefosine. It has been reported that these drugs act in distinct ways on parasite killing, which include the inhibition of metabolic pathways, cellular membrane damage and alteration of signal transductions, as recently reviewed [4]. However, the treatment of leishmaniasis is not ideal due to its toxicity and serious side effects, the high cost, and the prospect of the emergence of resistant parasites [5]. Therefore, new drug candidates, which are safer and more effective, are needed to improve the arsenal against this devastating disease.

**Figure 1:**
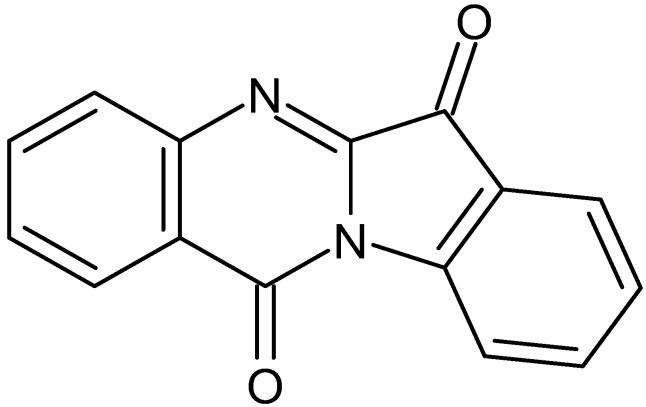
Chemical structure of tryptanthrin.

Tryptanthrin (Figure 1) is a naturally occurring compound that belongs to the alkaloid chemical class. It was identified in several botanical individuals including *Phaius mishmensis* [6]; *Couroupita guianensis* [7], *Polygonum tinctorium* Lour. [8], and [9] *Wrightia* spp. This indoloquinazoline alkaloid is described as a pharmacologically active agent against various illnesses [10]. Recently, tryptanthrin was reported as antiviral [11], antibacterial [7,12], antifungal [13], and antiprotozoal [14]. Regarding the antileishmanial activity, tryptanthrin and derivatives were active against axenic *Leishmania donovani* amastigotes [15]. Here, we investigated the mechanisms of action of this alkaloid against *L. amazonensis* and *L. infantum*. In addition, cytotoxicity and ADMET analysis were performed to provide first insights on tryptanthrin safety. 

## 2. Materials and Methods

### 2.1. Chemicals

Tryptanthrin, amphotericin B, annexin V-FITC kit, rhodamine 123, tetrazolium salt (MTT), monodansylcadaverine, and Dulbecco’s Modified Eagle Medium (DMEM) were obtained from Sigma-Aldrich (St. Louis, MO, USA). Dimethylsulfoxide (DMSO) was obtained from Dinâmica (Indaiatuba, SP, Brazil). Fetal bovine serum (FBS) was obtained from LGC Biotecnologia (Cotia, SP, Brazil).

### 2.2. Macrophage Viability Assay

The viability of the RAW 264.7 macrophage line was evaluated using the colorimetric method of tetrazolium salt (MTT) [16]. Initially, macrophages were cultured in DMEM supplemented with 10% FBS at 37 °C and 5% CO_2_ atmosphere. After sub-confluence was achieved (48 h), the cells were harvested, washed twice with cold PBS, and a macrophage suspension (10^6^ cells/mL) was prepared in a cold fresh medium. Aliquots of 100 µL were distributed into 96-well microplates, where the cells were allowed to adhere for 2 h. Subsequently, the alkaloid (1.97 to 252 µM, final concentrations) or amphotericin B (0.8 to 12.8 µM, final concentrations) were serially diluted and added to macrophage cultures. The cells were incubated for 48 h under the same conditions for cellular growth. After this period, the macrophage cultures were washed with PBS (phosphate saline buffer, pH 7.2) and re-incubated with 12 mM MTT solution for 3 h. Macrophage viability was determined spectrophotometrically at 570 nm (SpectraMax i3x, Molecular Devices, San Jose, CA, USA) after formazan crystal solubilization with DMSO. The results were expressed as mean values obtained from two independent experiments performed in duplicate. The CC_50_ was calculated based on the non-linear regression curves generated from the viability percentages. 

### 2.3. Anti-Promastigote Assay

*Leishmania* (*Leishmania*) *amazonensis* (MHOM/BR/75/Josefa) and *Leishmania* (*Leishmania*) *infantum* (MHOM/BR/1974/PP75) promastigotes were maintained as previously reported [17]. For the anti-promastigote assays, parasites were harvested at the late exponential phase (96 h) and washed twice with PBS. A parasite suspension was prepared (10^6^ cels/mL) from which 100 µL were transferred to 96-well plates containing several concentrations of a tryptanthrin solution (7.8 to 252 µM, final concentrations in PBHIL culture medium, and 1% DMSO). In addition, 1% DMSO-treated parasites were used as a negative control. The parasite cultures were incubated at 26 °C, and the growth inhibition effect was evaluated at the end of treatment (48 h) by optical density (OD) at 600 nm [18]. Percentages of inhibition were calculated considering the growth of controls (untreated parasites) as 100%. In addition, parasite viability at the end of treatment was determined after washing the treated cultures with PBS by centrifugation (2000 rpm/5 min) and re-incubation in a fresh medium (100 µL) for 96 h. The absence of growth indicated a leishmanicidal effect of the alkaloid. The minimal leishmanicidal concentration (MLC) was determined as the lowest concentration that completely abrogated the growth of parasites. The sub-leishmanicidal concentration (SLC) was defined as a two-fold dilution of MLC. The 50% (IC_50_) inhibitory concentrations were calculated by non-linear dose-response analysis. The results were expressed as mean values obtained from three independent experiments performed in duplicate.

### 2.4. Mitochondrial Membrane Potential (ΔΨ_m_)

The analysis of changes in ΔΨ_m_ of *L. amazonensis* and *L. infantum* promastigotes (2 × 10^6^ parasites) was carried out as previously reported [19], with some modifications. Initially, parasites were harvested as described above and then treated with IC_50_ values and a sub-leishmanicidal concentration (MLC/2) of tryptanthrin for 4 h at 26 °C. After the incubation period, the promastigote cultures were washed twice with PBS by centrifugation (2000 rpm/5 min), and the cell concentration was adjusted to 10^7^ parasites/mL in 200 µL PBS. The parasites were then transferred to 96-well microplates incubated in the presence of Rhodamine 123 (0.5 μg/mL) for 20 min in the dark at 26 °C. Finally, the fluorescence was measured at 485/528 nm (excitation/emission) using a spectrofluorometer (SpectraMax i3x, Molecular Devices, San Jose, CA, USA). Two independent experiments were performed in duplicate, using 1% DMSO-treated parasites as a negative control.

### 2.5. Assay for Autophagy

*L. amazonensis* and *L. infantum* promastigotes were harvested in the exponential phase (72 h), washed twice with PBS, and then resuspended in a fresh medium. Aliquots of 100 µL containing 2 × 10^6^ parasites were transferred to 96-well microplates containing 100 µL of tryptanthrin (IC_50_ values). Untreated parasites were used as a negative control. After a 24 h incubation period at 26 °C, both cultures (treated and untreated cells) were washed with PBS, resuspended in 100 µM monodansylcadaverine (MDC), and incubated for 1 h at 26 °C in the dark [20]. MDC is an autofluorescent autophagolysosome marker that specifically labels autophagic vacuoles in vivo and in vitro [21]. The parasite cultures were washed twice with PBS and fixed with 2% formaldehyde (20 min at room temperature). Finally, tryptanthrin-induced parasitic autophagy was determined fluorometrically at 335/460 nm (excitation/emission) using a SpectraMax i3x spectrofluorometer (Molecular Devices, San Jose, CA, USA).

### 2.6. Determination of Ergosterol Content in Promastigotes

The ergosterol content in *L. amazonensis* and *L. infantum* promastigotes was evaluated as described by Arthington-Skaggs et al. (1999), with modifications [22]. Parasites (10^5^ cells) were treated with tryptanthrin (IC_50_) for 18 h at 26 °C. After this time, the cells were washed with PBS and the wet cell weight was determined. Subsequently, 3 mL of alcoholic KOH solution was added, and the cells were subjected to vortex motion for 1 min. The samples were then incubated in a water bath (85 °C for 1 h) and cooled to room temperature. After this step, 1 mL of distilled water was added and the sterols were extracted by a liquid-liquid partition with 3 mL of cyclohexane. An aliquot of the organic phase was diluted (1:5) in ethanol, and this mixture was scanned (240–300 nm) on a SpectraMax i3x spectrofluorometer. The percentage of ergosterol was calculated as follows: % ergosterol + % 24(28)DHE = [(A_281,5_/290) × *F*]/cell wet weight, where *F*: ethanol dilution factor; % 24(28)DHE = [(A_240_/518) × *F*]/cell wet weight, where *F*: ethanol dilution factor; and % ergosterol = [% ergosterol + % 24(28)DHE] − % 24(28)DHE.

### 2.7. Annexin V-FITC/PI Dual Staining

*Leishmania* death by the apoptosis-like process was determined using an annexin V-FITC apoptosis detection kit as reported by Gabriel et al. (2019) with slight modifications [23]. First, *L. amazonensis* or *L. infantum* promastigotes in the exponential phase of growth were harvested, washed twice with PBS, and parasite suspensions (10^6^ parasites/mL) were prepared in a fresh medium containing tryptanthrin (IC_50_ value). Subsequently, the cultures were incubated for 18 h at 26 °C. Untreated parasites were used as a negative control. Amphotericin B was used as a reference drug and a positive control due to inhibition of the metacaspase pathway [24]. The annexin V-FITC/propidium iodide (PI) staining procedures were performed according to the manufacturer’s recommendations. Parasite death was determined by flow cytometry (BD FACSVerse™, BD Biosciences, San Jose, CA, USA), and data analysis was performed using Flowing Software 2.5.1 (Perttu Terho, Turku, Finland). The results were expressed as a percentage of positively stained cells relative to the total number of cells analyzed. PI-stained parasites indicate death by necrosis processes, while annexin- and annexin + PI-stained parasites indicate early and late apoptosis, respectively.

### 2.8. Macrophage Infection and Anti-Amastigote Assay

Cell suspensions containing RAW 264.7 macrophages were prepared and distributed into microplates as described above. *L. amazonensis* and *L. infantum* promastigotes were harvested at the stationary phase of growth (120 h). After the washing procedures, parasite suspensions were prepared in a fresh DMEM medium. The number of parasites was determined using a Neubauer chamber, and macrophage infection was then performed in a ratio of 10 parasites per host cell. Macrophage-parasite interaction occurred for 4 h at 37 °C and in an atmosphere of 5% CO_2_. The infected adherent cells were washed and then re-incubated for 24 h allowing promastigote-amastigote differentiation [25]. After this period, the infected macrophages were treated with increasing concentrations of tryptanthrin (16 to 201 µM) or amphotericin B (0.1 to 135 µM) for 48 h. The culture supernatants were collected at the treatment end to determine nitric oxide production by macrophages using the Griess reaction. Subsequently, the cells were washed with PBS before the addition of 200 µL of Schneider medium containing 0.05% SDS (sodium dodecyl sulfate). The addition of a low concentration of detergent induced controlled macrophage lysis, enabling the release of amastigotes and further differentiation into promastigotes [26,27]. Macrophage lysis was accompanied by light microscopy, and the released amastigotes were harvested and incubated in a fresh medium for 72 h at 26 °C to differentiate into promastigote forms. The MTT colorimetric assay was used to determine the viability of recovered promastigotes. It is noteworthy that only viable amastigotes can differentiate into promastigote forms. The IC_50_ was calculated based on the non-linear regression curves generated from the viability percentages.

### 2.9. Selectivity Index

The selectivity indexes (SI) for intracellular amastigotes of *L. amazonensis* and *L. infantum* were calculated by taking the ratio between the CC_50_ obtained for the host cell and the IC_50_ obtained for the parasites.

### 2.10. Prediction of ADMET by Computational Analysis

In silico ADMET (absorption, distribution, metabolism, excretion, and toxicity) analysis was performed using the PreADMET^®^ webserver (https://preadmet.bmdrc.kr/ (accessed on 31 March 2021)), in which pharmacokinetic properties (human intestinal absorption, plasma protein binding, CYP inhibition and substrate, and in vitro skin permeability), drug-likeness (Rule of 5), and toxicity (Ames test, carcinogenicity, and mutagenicity) were evaluated for tryptanthrin. Miltefosine was selected as a reference drug since the computational prediction of toxicity is based on experimental models that used the oral administration route.

### 2.11. Statistical Analysis

Statistical analysis was determined based on the Student’s *t*-test and two-way ANOVA with Sidak’s multiple comparisons test using the GraphPad Prism 8.0 software, considering *p* < 0.05 as significant.

## 3. Results

### 3.1. Tryptanthrin Cytotoxicity and Selectivity

Tryptanthrin cytotoxicity was evaluated against RAW 264.7 macrophages. The alkaloid showed a CC_50_ value of 465 µM (Table 1). After the anti-intracellular amastigote forms assays, the selectivity indexes of tryptanthrin were determined. We observed that the alkaloid was 6.2 and 4.0 times more toxic for *L. amazonensis* and *L. infantum* amastigotes than to host cells, respectively. 

### 3.2. Antileishmanial Activity

Tryptanthrin drastically inhibited promastigote proliferation in concentrations ≥32 µM (Figure S1). The minimal leishmanicidal concentration (MLC) was determined after re-incubation of tryptanthrin-treated promastigotes in fresh tryptanthrin-free culture media. The absence of growth indicated no viable cells in cultures exposed to concentrations ≥126 µM. The alkaloid displayed similar IC_50_ values against *L. amazonensis* and *L. infantum* promastigotes, and was 24 and 25 µM, respectively. Regarding the anti-intracellular amastigote activity, tryptanthrin was more effective against *L. amazonensis* amastigotes with an IC_50_ value of 75 µM (SI = 6.2). Despite the higher concentration necessary to reduce the number of *L. infantum* intracellular amastigotes (IC_50_ = 115 µM) by 50%, it was about four times inferior to that determined in the cytotoxic assays (CC_50_ = 465 µM). In addition, the alkaloid was not able to induce NO production by infected macrophages (data not shown). The effective concentrations against promastigote and intracellular amastigote forms of *L. amazonensis* and *L. infantum* are shown in Table 1.

### 3.3. Inhibition of Ergosterol Biosynthesis 

The effect of tryptanthrin on parasites’ ergosterol biosynthesis was evaluated after promastigote treatment with tryptanthrin (IC_50_ values for 18 h). Membrane sterols were extracted and quantified by spectrophotometry (Figure S2). We observed a slight decrease in the ergosterol content of *L. amazonensis* and *L. infantum* promastigotes treated with tryptanthrin (15% and 13%, respectively) when compared to controls. Likewise, the alkaloid poorly affected the content of 24(28)-dehydroergosterol (15% and 14%, respectively).

### 3.4. Mitochondrion Dysfunction 

**Figure 2:**
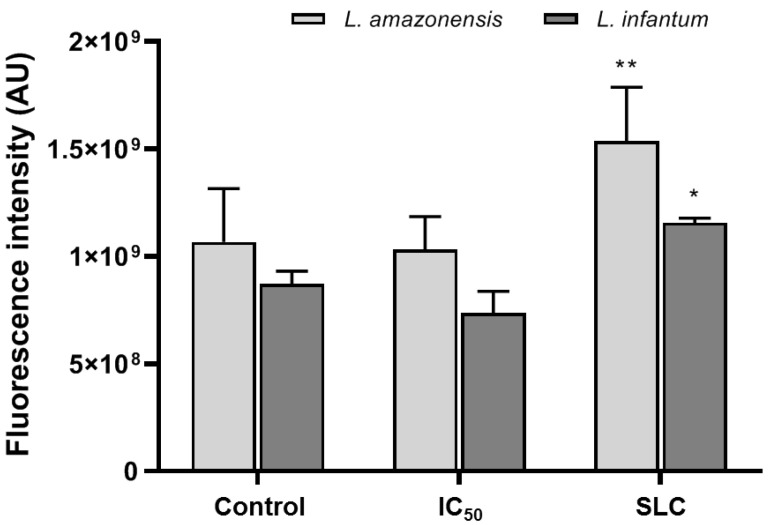
Effect of tryptanthrin on the mitochondrion membrane potential of *Leishmania* spp. Promastigote forms of *L. amazonensis* and *L. infantum* were treated with IC_50_ (11 and 8.0 µM, respectively) or SLC (63 µM, for both parasite species). A statistical analysis of the differences between mean values obtained for the experimental groups was done by two-way ANOVA with Sidak’s multiple comparisons test. Asterisks indicate treatment that was significantly different compared to the control, in which * *p* < 0.05 and ** *p* < 0.005. IC_50_, half-maximal inhibitory concentration; SLC, sub-leishmanicidal concentration (MLC/2).

Alterations in mitochondrion membrane potential (ΔΨ_m_) are a characteristic feature of apoptosis induction. Our results showed that tryptanthrin causes mitochondrion dysfunction on treated promastigotes by increasing ΔΨ_m_ (hyperpolarization). *L. amazonensis* and *L. infantum* promastigote forms treated with the sub-leishmanicidal concentration value (63 µM) showed an increase of 34% and 32% of their ΔΨ_m_ when compared to the untreated controls (Figure 2).

### 3.5. Autophagy Activity

**Figure 3:**
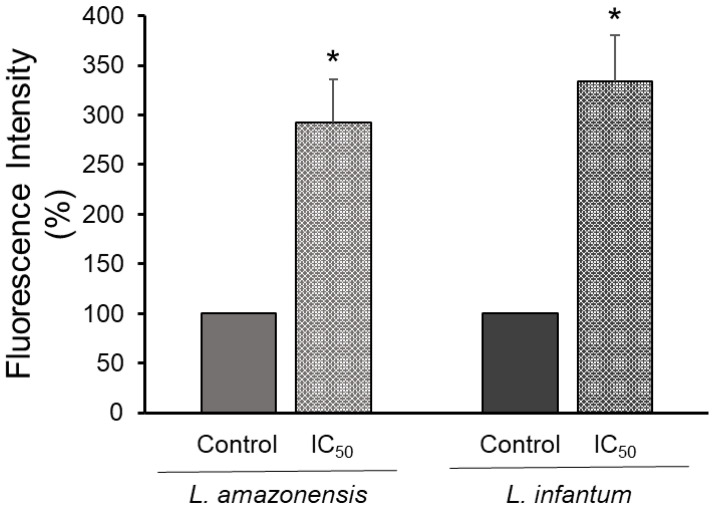
Effect of tryptanthrin on the autophagic activity of *Leishmania* spp. Promastigote forms of *L. amazonensis* and *L. infantum* were treated with IC_50_ values (11 and 8.0 µM, respectively) of tryptanthrin. The results are presented as the mean percentage relative to the control. Statistical analysis of the differences between mean values obtained for the experimental groups was done by Student’s *t*-test. The asterisks indicate significant differences (* *p* < 0.05) in the autophagic activity between treated parasites and their respective controls (100% activity).

Tryptanthrin’s effect on parasites’ viability and mitochondrion membrane hyperpolarization encourage us to investigate cellular death-related events to elucidate possible modes of action. Autophagy was investigated for treated *L. amazonensis* and *L. infantum* promastigotes after MDC staining procedures. MDC is a specific autofluorescent marker for autophagic vacuoles. After 18 h treatment (IC_50_ values), we observed an increase in fluorescence intensity of 192% and 234%, respectively, when compared to controls (untreated parasites) (Figure 3).

### 3.6. Leishmania Apoptosis-like Death

**Figure 4:**
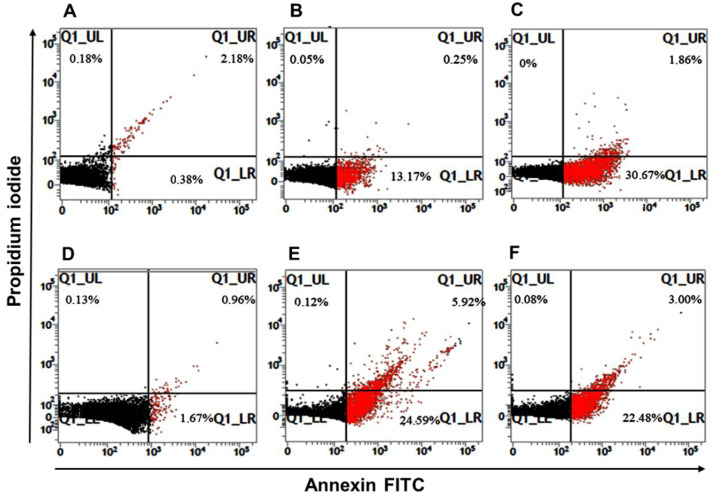
Representative flow cytometry dot-plots of apoptosis-like cell death of *Leishmania* spp. promastigotes induced by tryptanthrin. (**A**) Untreated *L. amazonensis*; (**B**) *L. amazonensis* treated with amphotericin B (IC50 = 0.68 µM); (**C**) *L. amazonensis* treated with tryptanthrin (IC50 = 11 µM); (**D**) Untreated *L. infantum*; (**E**) *L. infantum* treated with amphotericin B (IC50 = 0.86 µM); (**F**) *L. infantum* treated with tryptanthrin (IC50 = 8.0 µM).

Promastigote forms of *L. amazonensis* and *L. infantum* were treated with their respective IC_50_ values of tryptanthrin for 18 h, and cellular death was determined after annexin V-FITC/PI dual staining by flow cytometry (Figure 4). We observed that approximately 26% of *L. amazonensis* and *L. infantum* promastigotes undergo apoptosis (early + late apoptosis), respectively. The reference drug amphotericin B induced about 17% and 22% of parasites to apoptosis, respectively (Table 2).

### 3.7. ADMET Features of Tryptanthrin

To perform the computational pharmacokinetics and toxicological analysis of tryptanthrin, we used the PreADMET^®^ webserver. The alkaloid showed good bioavailability parameters, including intestinal absorption, cell permeability, plasma protein binding, and Lipinski’s Rule of Five (Table 3). In addition, the alkaloid is not an inhibitor of CYP enzymes and is just a weak substrate for CYP3A4. Nevertheless, tryptanthrin demonstrated mutagenic (Ames test) and carcinogenic potential (rodent models). These results were similar to those obtained for the reference drug miltefosine, the first oral drug approved for leishmaniasis treatment.

## 4. Discussion

*Leishmania* spp. susceptibility to the chemical class of alkaloids (natural or synthetic) has been demonstrated in the last decades [28,29]. Among them, N-based heterocyclic aromatic alkaloids have drawn attention due to their diverse pharmacological potential. Indeed, promising biological activities of quinoline and quinazoline alkaloids were recently reviewed [30,31]. Regarding the antiprotozoal activity, tryptanthrin and derivatives have been described as being effective against several parasite species. Onambele et al. (2015) demonstrated that the alkaloid displays remarkable antiplasmodial activity with IC_50_ values of 288 nM and 114 nM against chloroquine-sensitive and chloroquine-resistant strains of *Plasmodium falciparum*, respectively [32]. Previously, the antitrypanosomal activity of tryptanthrin and eleven other analogues were described against *Trypanosoma brucei* [33]. Here, we describe the indoloquinazoline alkaloid tryptanthrin as active against the dermotropic and viscerotropic species, *L. amazonensis* and *L. infantum*, respectively. The alkaloid was effective in the killing of both promastigote and intracellular amastigotes forms (Table 1). Concerning the anti-amastigote activity, we observed that the alkaloid did not stimulate NO production by infected macrophages. Indeed, tryptanthrin was described as an inhibitor of the expression of iNOS, the enzyme responsible for the synthesis of NO in macrophages [34]. Therefore, our results suggest that tryptanthrin eliminates intracellular amastigotes directly or by the activation of a nitrosative stress-independent mechanism.

Despite the use of tryptanthrins against other protozoal parasites, few reports described their antileishmanial activity. Bhattacharjee et al. (2002) screened twenty-seven tryptanthrin analogues for their anti-*Leishmania donovani* activity. The alkaloids exhibited remarkable IC_50_ values ranging from 16 ng/mL to 17 μg/mL against axenic amastigote forms. The authors suggested that the carbonyl groups at positions 5 and 6 of the tryptanthrin ring and electron transferability from a receptor are important for the observed antileishmanial activity [15]. In the search for a better understanding of how tryptanthrin leads parasites to death, we investigated some possible mechanisms of action. Previous reports showed that alkaloids can inhibit ergosterol’s biosynthesis of pathogenic yeasts [35,36]. In addition, the alkaloid magnoflorine affected the cell membrane by reducing the ergosterol content of *Trichophyton rubrum*, the etiological agent of Tinea pedis [37]. Medina et al. (2012) demonstrated that the glycoalkaloid tomatine induced some alterations in sterol metabolism of *L. amazonensis* in addition to ultrastructure damages and mitochondrion dysfunction [38]. These studies encourage us to evaluate a similar effect of tryptanthrin against *L. amazonensis* and *L. infantum*. Despite the fact that we observed that the alkaloid poorly inhibited total ergosterol content of both *Leishmania* species, it was able to cause mitochondrion dysfunction.

Alterations on ΔΨ_m_ have been correlated to elevated levels of reactive oxygen species (ROS) and mitochondrion damage. Taken together, all these events may culminate in the death of the parasites [39]. The isoquinoline alkaloid berberine chloride targeted the mitochondrion of *Leishmania* UR6 promastigotes. Berberine was described as an ROS inducer that increases mitochondrial superoxide levels. High levels of ROS led to mitochondrion membrane depolarization followed by depletion of ATP and apoptosis [40]. Similar results were previously described for *L. donovani* after exposure to camptothecin alkaloid [41]. Our results show that tryptanthrin affected *L. amazonensis* and *L. infantum* by mitochondrial membrane hyperpolarization. In addition, we observed that treated parasites increased autophagy activity, which is one feature of cells undergoing apoptosis.

The apoptotic effect of tryptanthrin on cancer cells is well documented [42,43,44]. Concerning its effects on trypanosomatid parasites, a study conducted by Frank et al. (2013) showed that C1, a thioamide-substituted imidazoquinolinone with a heterocyclic center similar to tryptanthrin, displayed anti-*Trypanosoma cruzi* activity (22.5 μM) through mitochondrion membrane depolarization and cytochrome *c* release into the cytoplasm. After 36 h exposure, C1 led about 58.7% and 7.7% of parasites to apoptosis (early + late apoptosis), respectively [45]. These results corroborate our findings, which demonstrate that tryptanthrin exerts its mode of action by the induction of *Leishmania* apoptosis.

Tryptanthrin displayed a cytotoxic effect against RAW 264.7 macrophages (CC_50_ value of 465 µM). It is worth noting that the alkaloid was 6.2 and 4.0 times more toxic for *L. amazonensis* and *L. infantum* intracellular amastigotes, respectively, than for the host cells. Despite the fact that a desirable SI >10 [46] was not achieved, we highlight the importance of testing multiple cells and complex organisms or using accurate analytical methods to generate evidence of the safe usage of tryptanthrin. In silico ADMET analysis is an interesting tool largely used for drug pharmacokinetics and toxicological predictions [47]. We performed a computational analysis of tryptanthrin and miltefosine using the PreADMET^®^ webserver. Tryptanthrin displayed high bioavailability scores, including intestinal absorption and plasma protein binding capacity, making it suitable for oral administration. In addition, tryptanthrin appears to be poorly metabolized in the liver, which may contribute to a low hepatotoxic effect. Despite that, tryptanthrin showed mutagenicity for the Ames test and carcinogenicity for murine models. Altogether, these results were similar to those obtained for the reference drug miltefosine, suggesting the promising usage of this alkaloid in the treatment of leishmaniasis. Recently, an ADMET analysis revealed good bioavailability parameters and suitable toxicological aspects of the antileishmanial alkaloids harmaline and harmine [48,49,50]. It is noteworthy that the drug-likeness of these alkaloids, including tryptanthrin, reinforces this chemical class as an interesting source of drug candidates.

## 5. Conclusions

Tryptanthrin exhibited anti-*L. amazonensis* and anti-*L. infantum* activities. Indeed, the alkaloid reduced the parasite load inside macrophages and could be considered as an interesting drug candidate against both clinical forms of leishmaniasis. The investigation of the tryptanthrin mechanism of action showed that the alkaloid might trigger parasite apoptosis. In addition, computational pharmacokinetics and toxicological evaluations of tryptanthrin indicated similar features to the reference drug miltefosine, including good oral bioavailability. Tryptanthrin can be used as a lead compound for the development of antileishmanial drugs.

## Figures and Tables

**Table 1:** Inhibition effects and cytotoxicity of tryptanthrin alkaloid. The results are expressed as a mean ± standard error of at least two independent experiments. Amphotericin B was used as a reference drug in the treatment of amastigote-infected macrophages.

Drugs	MØ		*L. amazonensis*					*L. infantum*			
	CC_50_ (µM)	MLC_Pro_ (µM)	SLC_Pro_ (µM)	IC_50Pro_ (µM)	IC_50Ama_ (µM)	SI	MLC_Pro_ (µM)	SLC_Pro_ (µM)	IC_50Pro_ (µM)	IC_50Ama_ (µM)	SI
Tryp	465 ± 31.05	126	63	11 ± 1.06	75 ± 11.63	6.2	126	63	8.0 ± 2.60	115 ± 2.79	4.0
Amph B	5.0 ± 0.89	n.d.	n.d.	0.68 ± 0.03	0.37 ± 0.14	13.5	n.d.	n.d.	0.86 ± 0.36	1.11 ± 0.21	4.5

Tryp, tryptanthrin; Amph B, amphotericin B; MØ, RAW 264.7 macrophages; MLC, minimal leishmanicidal concentration; SLC_Pro_, sub-leishmanicidal concentration (MLC/2); IC_50Pro_, 50% inhibitory concentration of the drug against promastigote forms; IC_50Ama_, 50% inhibitory concentration of the drug against intracellular amastigote forms; SI, selectivity index (CC_50_/IC_50Ama_). n.d., not determined.

**Table 2:** Flow cytometry analysis of *L. amazonensis* and *L. infantum* promastigotes treated with tryptanthrin. The results are demonstrated as the percentage of propidium iodide (necrosis), annexin FITC-V (early apoptosis), and propidium iodide/annexin FITC-V (late apoptosis) positive parasites.

Drugs	*L. amazonensis*				*L. infantum*			
	PI	AV	PI/AV	Total Apoptosis (AV + PI/AV)	PI	AV	PI/AV	Total Apoptosis (AV + PI/AV)
Tryp	0.79 ± 0.07	24.73 ± 5.94	1.35 ± 0.51	26.08	0.06 ± 0.02	22.90 ± 0.42	2.80 ± 0.20	25.70
Amph B	0.09 ± 0.04	15.98 ± 2.81	0.88 ± 0.02	16.86	0.09 ± 0.03	17.06 ± 7.53	4.81 ± 1.11	21.87
Control	0.23 ± 0.04	0.25 ± 0.13	2.5 ± 0.31	2.98	0.11 ± 0.02	1.42 ± 025	0.64 ± 0.32	2.06

Tryp, tryptanthrin; Amph B, amphotericin B; AV, annexin FITC-V; PI, propidium iodide.

**Table 3:** In silico predicted pharmacokinetic and toxicological parameters of tryptanthrin and miltefosine.

Drug	ADME							Druglikeness	Toxicity			
	SP ^a^ (log Kp, cm/h)	HIA ^b^ (%)	PPB ^c^ (%)	MDCK ^d^ (nm/s)	CYP ^e^ Inhibitor	CYP ^f^ Substrate	BBB ^g^ [Brain]/[Blood]	Rule of 5 ^h^	Ames Test ^i^	CarR ^j^	CarM ^j^	hERG ^k^
Tryp	−3.8	97.4	85.8	166.2	non	CYP3A4 (weakly)	1.94	Suitable	Mutagen	Positive	Positive	Medium risk
MTF	−0.80	98.1	86.2	43.4	CYP2D6	CYP3A4	0.13	Suitable	Mutagen	Positive	Negative	Low risk

Tryp, tryptanthrin; MTF, miltefosine; ^a^ In vitro skin permeability (transdermal delivery); ^b^ Human intestinal absorption data are the sum of bioavailability and absorption evaluated from ratio of excretion or cumulative excretion in urine, bile and feces; ^c^ In vitro plasma protein binding; ^d^ In vitro MDCK (Madin-Darby canine kidney) reliable cell model for the prediction of oral drug absorption; ^e^ In vitro inhibition of cytochrome P450 isoforms; ^f^ In vitro substrate of cytochrome P450 isoforms; ^g^ In vivo blood-brain barrier penetration expressed as the ratio between steady-state concentration of radiolabeled compounds in brain and peripheral blood; ^h^ Theoretical oral bioavailability based on the Lipinski’s Rule of Five (Lipinski, 2004); ^i^ Method to test mutagenicity of a compound based on its ability to cause a reversion on growth of histidine-dependent *Samonella typhimurium* in a histidine-free medium; ^j^ Rodent carcinogenicity two year assay of rat (CarR) and mouse (CarM) by backward elimination and Rprop neural net method; ^k^ Inhibition of the human Ether-a-go-go Related Gene (hERG) potassium channel.

## Data Availability

The dataset analyzed during the current study is available from the last author on reasonable request.

## Supplementary Materials

The following supporting information can be downloaded at: https://www.mdpi.com/article/10.3390/tropicalmed7060112/s1, Figure S1: Antileishmanial activity of tryptanthrin; Figure S2: Ergosterol content of *L. amazonensis* and *L. infantum* promastigotes treated with tryptanthrin.
