# Supplementary material for: The Natural Alkaloid Tryptanthrin Induces Apoptosis-like Death in Leishmania spp
Source: Trop Med Infect Dis. 2022 Jun 20;7(6):112. doi: 10.3390/tropicalmed7060112 (PMC9231190; doi:10.3390/tropicalmed7060112)
Supplement: Supplementary file 1 [file tropicalmed-07-00112-s001.zip › tropicalmed-1751735-supplementary.pdf]

Supplementary

The Natural Alkaloid Tryptanthrin Induces Apoptosis-like Death in *Leishmania* spp.

Andreza R. Garcia [1,†], Yasmin P. G. Silva-Luiz [2,†], Celuta S. Alviano [3], Daniela S. Alviano [3], Alane B. Vermelho [3] and Igor A. Rodrigues [4,*]

[1] Graduate Program in Pharmaceutical Sciences, School of Pharmacy, Federal University of Rio de Janeiro, Rio de Janeiro 21941-902, Brazil; deh.raposo@yahoo.com.br

[2] Graduate Program in Science (Microbiology), Institute of Microbiology, Federal University of Rio de Janeiro, Rio de Janeiro 21941-902, Brazil; yasminpaulaluiz@gmail.com

[3] Department of General Microbiology, Institute of Microbiology, Federal University of Rio de Janeiro, Rio de Janeiro 21941-902, Brazil; alviano@micro.ufrj.br (C.S.A.); danialviano@micro.ufrj.br (D.S.A.); abvermelho@micro.ufrj.br (A.B.V.)

[4] Department of Natural Products and Food, School of Pharmacy, CCS, Federal University of Rio de Janeiro, Rio de Janeiro 21941-902, Brazil

* Correspondence: igor@pharma.ufrj.br

† These authors contributed equally to this work.

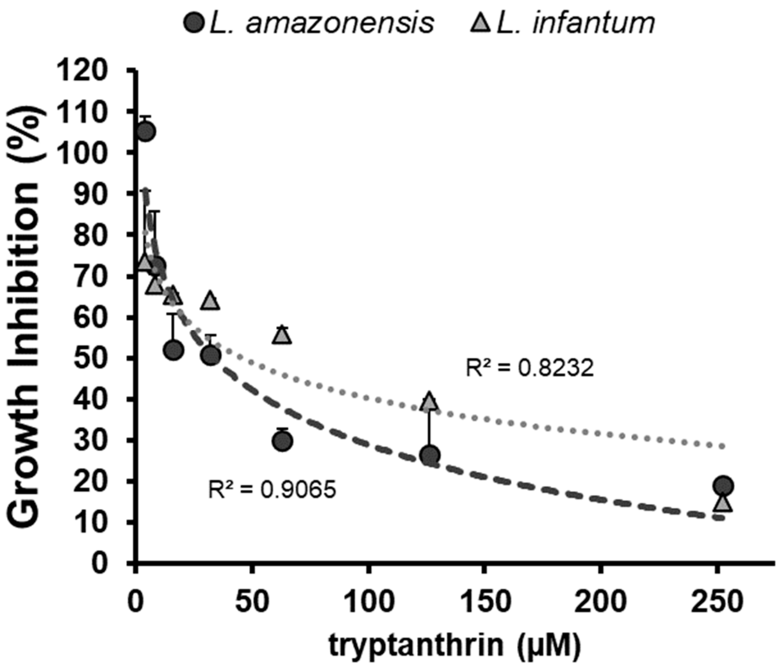

Figure S1. Antileishmanial activity of tryptanthrin. Promastigote forms of *L. amazonensis* (●) or *L. infantum* (▲) were treated with the alkaloid for 48 h. The parasite growth is expressed as a percentage of the OD compared with that of the control. The IC_{50} values were calculated by nonlinear regression analysis.

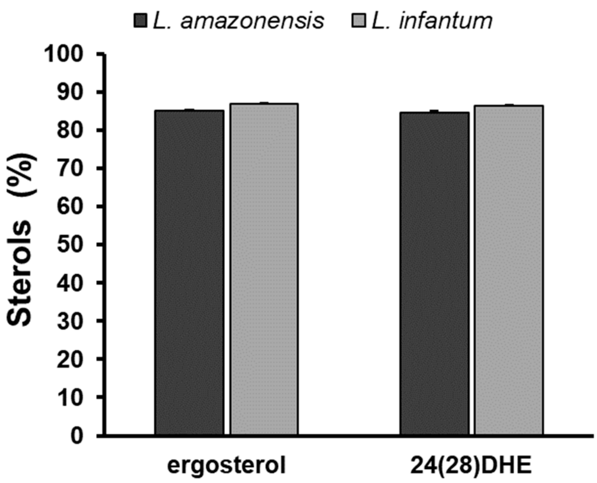

Figure S2. Ergosterol content of *L. amazonensis* and *L. infantum* promastigotes treated with tryptanthrin. The results are presented as the mean percentage relative to the control. Statistical analysis of the differences between mean values obtained for the experimental groups was done by Student's *t*-test. No significant differences were observed in the ergosterol content between treated parasites.
